# Highly Sensitive and Selective Defect WS_2_ Chemical Sensor for Detecting HCHO Toxic Gases

**DOI:** 10.3390/s24030762

**Published:** 2024-01-24

**Authors:** Zhen Cui, Hanxiao Wang, Kunqi Yang, Yang Shen, Ke Qin, Pei Yuan, Enling Li

**Affiliations:** 1 School of Science, Xi’an University of Technology, Xi’an 710054, China; 2220920058@stu.xaut.edu.cn (H.W.); 2210920050@stu.xaut.edu.cn (K.Y.); shenyang@xaut.edu.cn (Y.S.); 2220920069@stu.xaut.edu.cn (K.Q.); 2220920057@stu.xaut.edu.cn (P.Y.); 2 School of Automation and Information Engineering, Xi’an University of Technology, Xi’an 710048, China

**Keywords:** defect WS_2_ chemical sensors, sensitivity, selectivity, toxic gases

## Abstract

The gas sensitivity of the W defect in WS_2_ (V_W_/WS_2_) to five toxic gases—HCHO, CH_4_, CH_3_HO, CH_3_OH, and CH_3_CH_3_—has been examined in this article. These five gases were adsorbed on the V_W_/WS_2_ surface, and the band, density of state (DOS), charge density difference (CDD), work function (*W*), current–voltage (I–V) characteristic, and sensitivity of adsorption systems were determined. Interestingly, for HCHO-V_W_/WS_2_, the energy level contribution of HCHO is closer to the Fermi level, the charge transfer (*B*) is the largest (0.104 e), the increase in *W* is more obvious than other adsorption systems, the slope of the I–V characteristic changes more obviously, and the calculated sensitivity is the highest. To sum up, V_W_/WS_2_ is more sensitive to HCHO. In conclusion, V_W_/WS_2_ has a great deal of promise for producing HCHO chemical sensors due to its high sensitivity and selectivity for HCHO, which can aid in the precise and efficient detection of toxic gases.

## 1. Introduction

The majority of volatile organic compounds (VOCs) such as HCHO, CH_4_, CH_3_CHO, CH_3_OH, and CH_3_CH_3_ are hazardous to human health and safety, the environment, and cause cancer [1]. In order to identify harmful gases and lessen their damage, we need devices. Chemical sensors are commonly used to detect and analyze chemical substances and can measure the existence and specific concentration of experimental substances. Researchers have been continuously exploring and innovating chemical sensors to address the issue of harmful gas detection and monitoring in various domains.

In the current period of rapid scientific and technological advancement, chemical sensors are progressively becoming essential instruments across a wide range of fields. These sensors have the ability to detect sensitive signals and accurately identify chemicals, thus affecting our life, health, and environment. For instance, the use of chemical sensors is significant in the field of environmental protection. A number of toxic gases, including CO and SO_2_, are present in the atmosphere and are extremely dangerous to both human health and the environment. Scientists can track and identify the concentration of these dangerous gases in real time and take appropriate action to protect the environment by inventing high-performance chemical sensors. Furthermore, the use of chemical sensors in the industrial sector is very important. Consider a chemical plant as an example. During the production process, several hazardous gases are emitted, including H_2_S and NH_3_. High-performance chemical sensors allow workers to detect and identify the presence of dangerous gases in real time, allowing them to avoid potential hazards that could endanger their health. Furthermore, the medical industry is also involved in the application of chemical sensors. For instance, certain harmful gases, such as NO, will be created during respiratory therapy and will negatively affect the health of patients. Medical personnel can monitor the concentration of these dangerous gases in real time and take appropriate action to safeguard patients’ respiratory systems by employing chemical sensors with excellent sensitivity and selectivity. 

To put it succinctly, chemical sensors are crucial for the identification and tracking of toxic gases. Continuous research and innovation can enhance the dependability, sensitivity, and accuracy of chemical sensors. 

Chemical sensors have been designed using nanomaterials on a large scale in recent years due to the advancement of nanotechnology [2,3,4]. Chemical sensors’ performance has been greatly enhanced using nanomaterials, primarily in the following areas: Increasing specific surface area: in comparison to conventional materials, nanomaterials have a higher specific surface area per unit mass or volume. As a result, nanomaterials can offer more active sites and improve their interaction with target molecules, increasing the sensitivity of the sensor [5]. Enhance conductivity: nanomaterials often exhibit high levels of thermal and electrical conductivity, which helps to enhance sensors’ response times and signal conduction efficiency. This holds great significance for instantaneous monitoring and prompt reaction to specific compounds, particularly in the domains of environmental and medical monitoring. Introducing new features: nanomaterials exhibit physical and chemical properties distinct from large-scale materials due to the size effect and quantum effect. New properties, such as optics, electricity, and magnetism, can be added to nanomaterials with great care to improve the sensor’s selectivity and reactivity to target molecules. Boost stability and durability: the high surface area and unique structure of nanomaterials contribute to the sensors’ increased stability and durability. Realizing multifunctional design: By mixing several nanomaterial types, chemical sensors with multifunctional capabilities can be designed thanks to the multifunctional nature of nanomaterials. This multifunctional architecture enhances the sensor’s overall performance by simultaneously detecting numerous target molecules. As a result, the introduction of nanomaterials opens up new avenues for chemical sensor design. 

Chemical sensors have advanced quickly in the last few years, but there are still a lot of restrictions. Certain chemical sensors can produce erroneous measurement findings due to cross-sensitivity to toxic gases, which occurs when other gases interfere with the sensor [6]. Different chemical sensors have varying degrees of stability and accuracy, and some are more sensitive to environmental factors like humidity, temperature, and pressure, which require calibration and adjustment [7,8,9]. High sensitivity and selectivity are necessary for the sensor to enable effective monitoring, and high performance is required for the detection of toxic gases. Thus, in order to address the needs of toxic gas detection and monitoring in many domains, we should keep researching and developing chemical sensors [10,11,12].

High-performance gas-sensitive materials must be developed in order to achieve the sensitive detection of toxic gases [13]. Transition metal dichalcogenides [14,15,16] have a number of superior properties in the realm of chemical sensors, which makes them perfect building blocks for the production of effective sensors. Through the deft application of these properties, we can achieve tremendous gains in sensor performance and design [17,18,19]. Cui researched how the photoelectric properties of WS_2_ and defect for the WS_2_ changed when it was adsorbed by CO, NH_3_, NO, and NO_2_ gas molecules in 2022 [11]. They came to the conclusion that WS_2_ is useful for toxic gas detection and gas sensing. WS_2_ has a large surface area, active sites, high sensitivity, and controllability, which are beneficial to molecular adsorption and interaction with target molecules, and can achieve highly selective recognition of specific molecules and improve the sensitivity and response speed of the sensor. 

On the surface of WS_2_ and the W defect for WS_2_ V_W_/WS_2_, we adsorbed HCHO, CH_4_, CH_3_HO, CH_3_OH, and CH_3_CH_3_, and we computed their electronic properties and work function (*W*). We selected the V_W_/WS_2_ adsorption systems to further compute the I–V characteristic and sensitivity since the results were consistent that the electronic properties and *W* of HCHO-WS_2_ and HCHO-V_W_/WS_2_ changed most obviously in the WS_2_ adsorption systems and the V_W_/WS_2_ adsorption systems [20,21,22]. We discovered that V_W_/WS_2_ exhibited excellent sensitivity and selectivity for HCHO. Due to its high sensitivity, V_W_/WS_2_ is able to identify toxic gas with a very low concentration of HCHO [23]. Because HCHO is frequently damaging to human health at low concentrations, this is crucial for safeguarding both the environment and people. Second, selectivity refers to V_W_/WS_2_’s ability to recognize variations in various gases and solely react to the toxic gas (HCHO) [24]. Due to these benefits, V_W_/WS_2_ is a valuable tool for environmental monitoring, industrial safety, and personal safety. It also makes it easier to recognize and manage toxic gases, which is particularly useful for HCHO detection [25].

## 2. Computational Methods

We calculated the density functional theory (DFT) by using the Vienna ab initio simulation software package 5.4.4 (VASP). The exchange–correlation interaction and the electron–ion interaction are described, respectively, by the extended gradient approximation of Perdew Burke Ernzerhof (PBE) and the projection-enhanced wave approach. In order to assure system stability, we built a 4 × 4 × 1 supercell model and a vacuum layer of 20 Å was added in the z direction for WS_2_, defect WS_2_, and the adsorption systems. For both structural and electronic structure optimization calculations, the sampling grid in the K space of the Brillouin zone is 9 × 9 × 1, the energy convergence criterion is 10^−7^ eV, the ideal convergence threshold is to guarantee that the force acting on atoms is less than 10^−3^ eV/Å, and the cutoff energy is set to 500 eV.

The adsorption energy (*E*_ab_) between molecules was further evaluated. The energy generated when free monolayers combine to form adsorption systems is known as the *E*_ab_, and it is expressed as [26,27,28,29]:

(1) $$E_{\mathrm{abs}}=E_{\mathrm{adsorbedsystem}}-\left(E_{\mathrm{monolayer}}+E_{\mathrm{targetmolecule}}\right)$$

The energy released during the construction of the adsorption system is represented by the symbol *E*_abs_ in the above equation. The total energy of the adsorption systems is also indicated by the symbol *E*_adsorbedsystem_. Additionally, the total energy of V_W_/WS_2_ and gas molecules is indicated by *E*_monolayer_ and *E*_targetmolecule_. Lastly, the unit area under consideration is indicated by the sign *S*.

By utilizing computed differences in charge density difference (CDD) [30] and charge transfer (*B*) [31], we can analyze the transfer of charges between the adsorption systems. This formula can be expressed as follows:

(2) $$\Delta \rho =\rho_{h}-\rho_{1}-\rho_{2}$$

By employing computed variations in charge density (*ρ*_h_, *ρ*_1_, and *ρ*_2_), we can examine the *B* mechanisms between the V_W_/WS_2_ and gas molecules. Here, *ρ*_h_ represents the charge density of the adsorption systems, while *ρ*_1_ and *ρ*_2_ correspond to the charge densities of the V_W_/WS_2_ and gas molecules.

We studied the I–V characteristic of the adsorption systems, which was obtained by calculating the current at different bias voltages. The current at a specific bias voltage can be obtained using the following equation [32]:

(3) $$I=\frac{e}{h}\int_{-\infty }^{+\infty }T(E;V_{b})[f_{L}(E;E_{F}^{L}-V_{b}/2)-f_{R}(E;E_{F}^{R}+V_{b}/2)]dE$$

where $f_{L}(E;E_{F}^{L}-V_{b}/2)$, $E_{F}^{L}$, $f_{R}(E;E_{F}^{R}-V_{b}/2)$, and $E_{F}^{R}$ are the Fermi energy level and Fermi distribution at the equilibrium state of the left and right electrodes, respectively. $T(E;V_{b})$ is the transmission probability function spectrum for electrons.

Lastly, we used the following formula to evaluate the adsorption systems’ sensitivity [33]:

(4) $$S=(G-G_{0})/G_{0}\times 100\%$$

where *G* is the conductance of adsorption systems, and *G*_0_ is the conductance of V_W_/WS_2_.

We used Vienna ab initio simulation package (VASP) software to calculate the electronic properties of the systems, such as energy band, density of state (DOS), CDD, and *B*, and used Nanodcal 2023A software to calculate the electrical characteristics of I–V [34,35].

## 3. Results and Discussion

**Figure 1:**
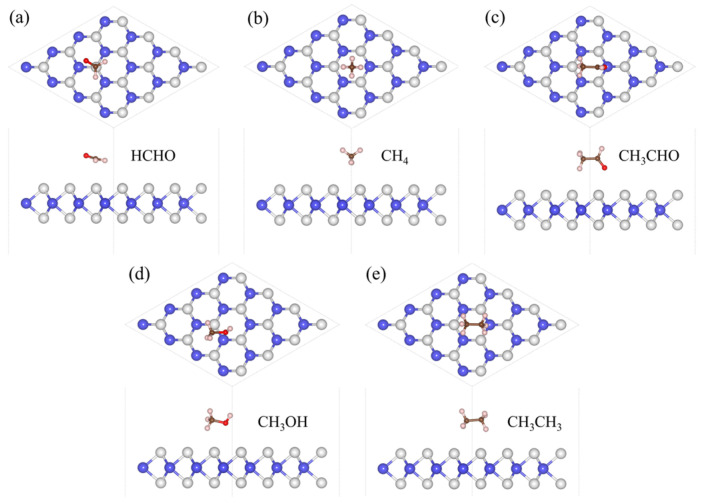
The most stable adsorption configurations of (**a**) HCHO, (**b**) CH_4_, (**c**) CH_3_CHO, (**d**) CH_3_OH, and (**e**) CH_3_CH_3_ adsorbed on WS_2_. The white, blue, brown, pink, and red spheres represent S, W, C, H, and O atoms, respectively.

**Figure 2:**
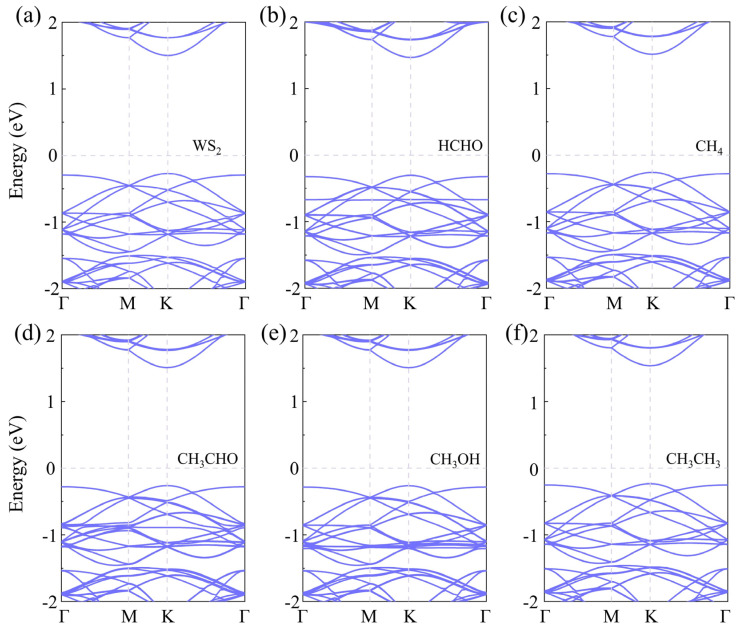
The band of (**a**) WS_2_, (**b**) HCHO-WS_2_, (**c**) CH_4_-WS_2_, (**d**) CH_3_CHO-WS_2_, (**e**) CH_3_OH-WS_2_, and (**f**) CH_3_CH_3_-WS_2_.

**Figure 3:**
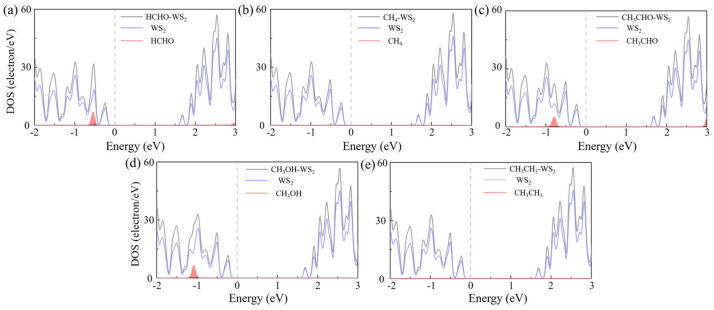
The DOS of (**a**) HCHO, (**b**) CH_4_, (**c**) CH_3_CHO, (**d**) CH_3_OH, and (**e**) CH_3_CH_3_ adsorbed on WS_2_.

**Figure 4:**
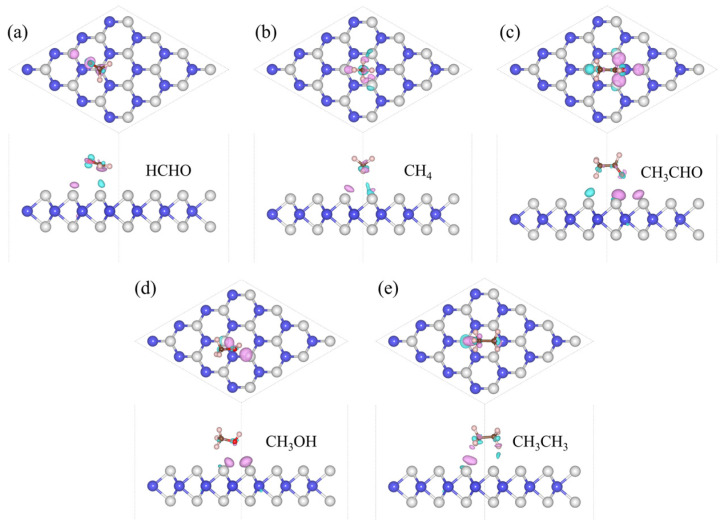
The CDD of (**a**) HCHO, (**b**) CH_4_, (**c**) CH_3_CHO, (**d**) CH_3_OH, and (**e**) CH_3_CH_3_ adsorbed on WS_2_ with the isosurface value of 3 × 10^−4^ e Å^−1^. The W and S atoms are colored in blue and grey, the sky-blue area denotes the accumulation of electrons, and the purple area denotes the depletion of electrons.

The focus of our research is toxic gases, and HCHO, CH_4_, CH_3_CHO, CH_3_OH, and CH_3_CH_3_ are the most common toxic gases. There is still a technical gap in the field of detection. So, we adsorbed five gas molecules, HCHO, CH_4_, CH_3_CHO, CH_3_OH, and CH_3_CH_3_, on WS_2_ and optimized their structures. The adsorption distance (*H*) of the adsorption systems before optimization is 3 Å, the optimized *H* values are 2.94 Å, 2.80 Å, 2.78 Å, 2.49 Å, and 2.52 Å in Table 1, respectively. We also calculated the *E*_ab_ of the adsorption systems and determined the most stable model, as shown in Figure 1. Next, the energy bands of the adsorption systems were calculated, as shown in Figure 2. We can see that the energy bands have hardly changed, so the adsorption of gas molecules has little effect on the electronic properties of WS_2_. Gas molecules are physically adsorbed. In order to further investigate the physical adsorption of gas molecules, we calculated the DOS; the total DOS of the adsorption system is shown by the gray line in Figure 3, the DOS of WS_2_ is represented by the blue line. Compared with the DOS of WS_2_, the adsorption systems show a DOS contribution of gas molecules near the Fermi level and the DOS of gas molecules is represented by the red line. The DOS of CH_4_ and CH_3_CH_3_ disappeared near the Fermi level. The DOS of CH_3_OH and CH_3_CHO is further away from the Fermi level, while the DOS of HCHO is closest to the Fermi level. Therefore, HCHO adsorption has the greatest impact on the electronic properties near the Fermi level compared to other gas adsorption. Figure 4 is the CDD we calculated; the sky-blue area denotes the charge accumulation, and the purple area denotes the charge depletion. The electric field is usually formed by the charge accumulation, and the charge depletion leads to the flow and transport of charges, which in turn affects the electrical characteristics of materials. There is a difference between the energy level of the adsorbed substance and the energy level of the surface, and the movement of electrons leads to the resonance of electrons or the change in local electron density, thus causing *B* in the adsorption systems. We can see that there is *B* between the gas molecule and WS_2_. In Table 1, combined with the *B* of the adsorption systems (0.107 e, 0.092 e, 0.088 e, 0.091 e, and 0.089 e, respectively), the *B* of the HCHO-WS_2_ system is the largest, which is consistent with DOS. Next, we calculated the *W* of the WS_2_ adsorption systems, which are 5.72 eV, 5.53 eV, 5.01 eV, 5.15 eV, and 5.53 eV, respectively. Compared with the *W* of WS_2_ (5.39 eV), the *W* of HCHO-WS_2_ increased the most significantly, while the *W* of CH_3_CHO-WS_2_ and CH_3_OH-WS_2_ decreased. The *W* of CH_4_-WS_2_ and CH_3_CH_3_-WS_2_ show a slight increase. So, when detecting gas, WS_2_ is more likely to react with HCHO. In summary, WS_2_ has higher sensitivity and selectivity towards HCHO.

**Figure 5:**
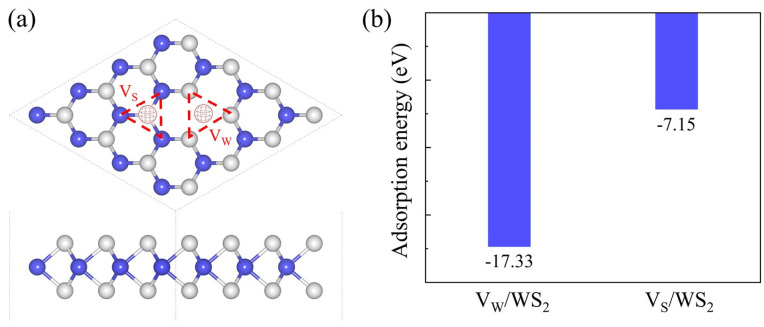
The (**a**) configuration and (**b**) adsorption energy of V_W_/WS_2_ and V_S_/WS_2_, the W and S atoms are colored in blue and grey.

**Figure 6:**
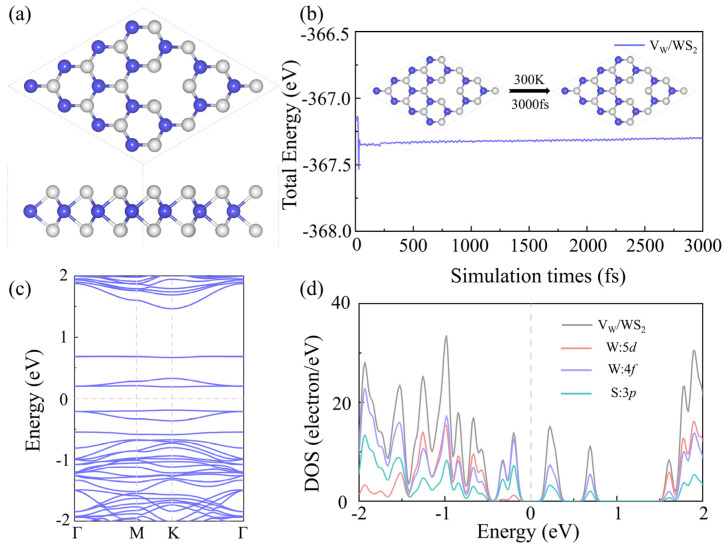
The (**a**) configuration, (**b**) graph of total energy and simulation time, (**c**) band structure, and (**d**) DOS of V_W_/WS_2_, the W and S atoms are colored in blue and grey.

An important research object in the study of 2D materials is vacancy defects. By adding vacancy defects in certain ways, scientists can investigate how defects affect electrical conductivity, optical absorption, electronic structure, and other aspects. Researchers can gain a deeper understanding of the relationship between a material’s structure and properties, as well as expand the potential applications of 2D materials in areas like catalysis, sensing, and electrical devices through vacancy defects. Based on the WS_2_ model, we removed the W atom to construct V_W_/WS_2_, and similarly removed the S atom to construct S defect WS_2_ (V_S_/WS_2_), the model diagram of which is displayed in Figure 5a. Next, we computed the *E*_ad_ of these two structures after fully relaxing them. The *E*_ad_ of V_W_/WS_2_ is −17.33 eV, and that of V_S_/WS_2_ is −7.15 eV, as Figure 5b illustrates. We decided to focus our subsequent research on V_W_/WS_2_ since the more negative adsorption energy it has, the more stable it becomes [36]. To gain more insight into this structure’s thermal stability, we computed the ab initio molecular dynamics (AIMD) at 300 K [37,38,39]. V_W_/WS_2_ exhibits thermal stability, as evidenced by Figure 6b, where the total energy tends to stabilize across the simulation duration and the crystal structure is free of deformation and bond breaking. We computed the band structure [3] and DOS of V_W_/WS_2_ in order to comprehend its electronic properties. V_W_/WS_2_ is a direct band gap semiconductor with a band gap value of 0.379 eV, as can be seen in the band diagram in Figure 6c. The Fermi level is situated between the conduction band and the valence band, V_W_/WS_2_ is a semiconductor, and the 4f level of the W atom supplies the valence band maximum and conduction band minimum, which correlates to the energy band, as can be seen in the DOS diagram of Figure 6d. 

**Figure 7:**
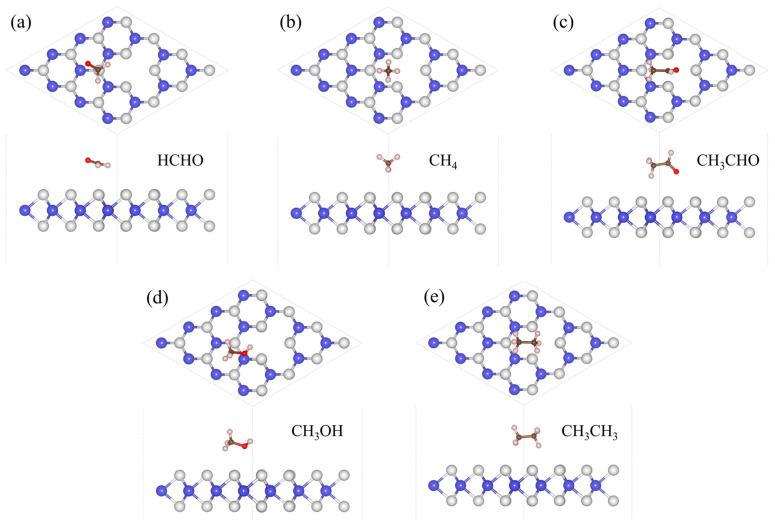
The most stable adsorption configurations of (**a**) HCHO, (**b**) CH_4_, (**c**) CH_3_CHO, (**d**) CH_3_OH, and (**e**) CH_3_CH_3_ adsorbed on V_W_/WS_2_, the W and S atoms are colored in blue and grey.

**Figure 8:**
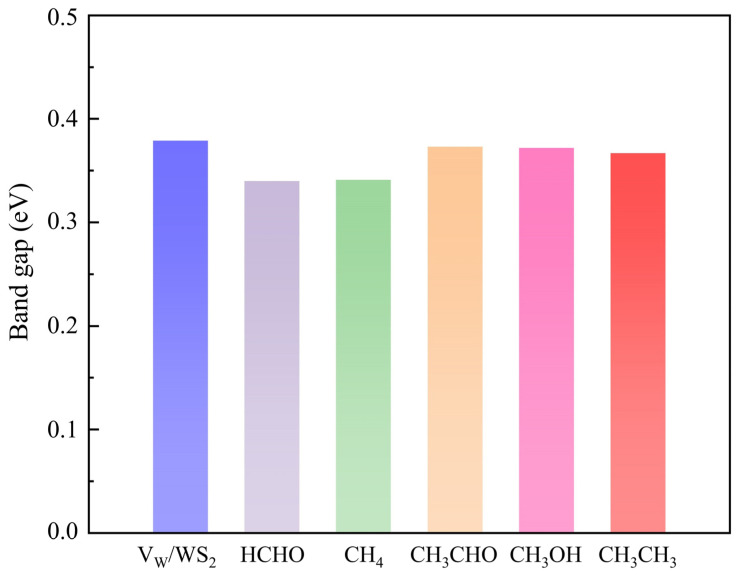
The band gap of HCHO, CH_4_, CH_3_CHO, CH_3_OH, and CH_3_CH_3_ adsorbed on V_W_/WS_2_.

**Figure 9:**
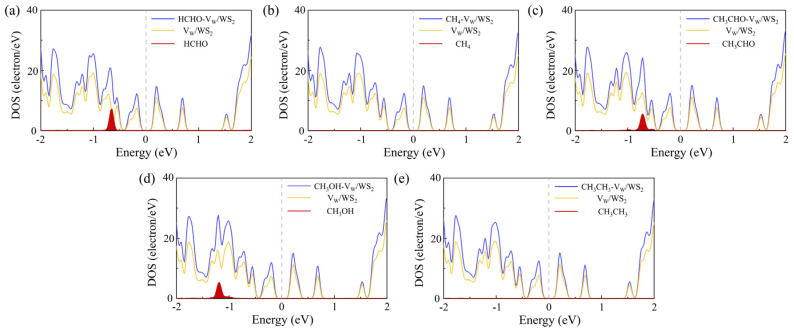
The DOS of (**a**) HCHO, (**b**) CH_4_, (**c**) CH_3_CHO, (**d**) CH_3_OH, and (**e**) CH_3_CH_3_ adsorbed on V_W_/WS_2_.

**Figure 10:**
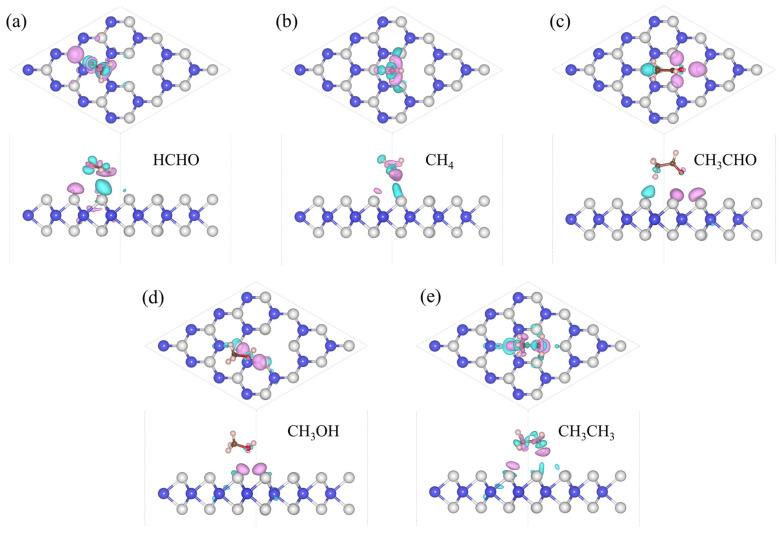
The CDD of (**a**) HCHO, (**b**) CH_4_, (**c**) CH_3_CHO, (**d**) CH_3_OH, and (**e**) CH_3_CH_3_ adsorbed on V_W_/WS_2_. The W and S atoms are colored in blue and grey, the sky-blue area denotes the accumulation of electrons, and the purple area denotes the depletion of electrons. The value of the isosurface is set to 3 × 10^−4^ e Å^−1^.

The *H* of the adsorption systems before optimization is 3 Å. In Table 2, the most stable model (the *H* values are 2.92 Å, 2.79 Å, 2.65 Å, 2.67 Å, and 2.51 Å, respectively) for V_W_/WS_2_ to adsorb the five toxic gases—HCHO, CH_4_, CH_3_CHO, CH_3_OH, and CH_3_CH_3_—is depicted in Figure 7 [11,40]. The band gap values of V_W_/WS_2_ and the five adsorption systems are displayed in Figure 8 [41]. We can see that compared with V_W_/WS_2_ the band gap undergoes certain changes after adsorbing gas molecules. The DOSs of adsorption systems are displayed in Figure 9. Compared with other gas molecules, due to the structure and electronic configuration of HCHO, its oxygen atom may introduce more electrons, and some special electronic states may be introduced near the Fermi level, resulting in the formation of a local electronic state near the Fermi level. The DOS of HCHO is closer to the Fermi level and its contribution is greater near the Fermi level. The DOSs of CH_4_ and CH_3_CH_3_ disappear near Fermi level, while the DOSs of CH_3_CHO and CH_3_OH are far away from Fermi level and their contribution is smaller. Therefore, V_W_/WS_2_ has high sensitivity and selectivity for HCHO. The CDD of the adsorption systems is shown in Figure 10. We can see the accumulation and dissipation of charge in the adsorption systems, which shows that the conductivity of V_W_/WS_2_ has changed to some extent after adsorbing gas molecules. In order to describe which gas molecules V_W_/WS_2_ is most sensitive to, we calculated *B*. As shown in Table 2, HCHO-V_W_/WS_2_ has the highest *B*, which shows that the conductivity of HCHO-V_W_/WS_2_ has the most obvious change, and V_W_/WS_2_ has high sensitivity and selectivity to HCHO [12,41,42,43].

**Figure 11:**
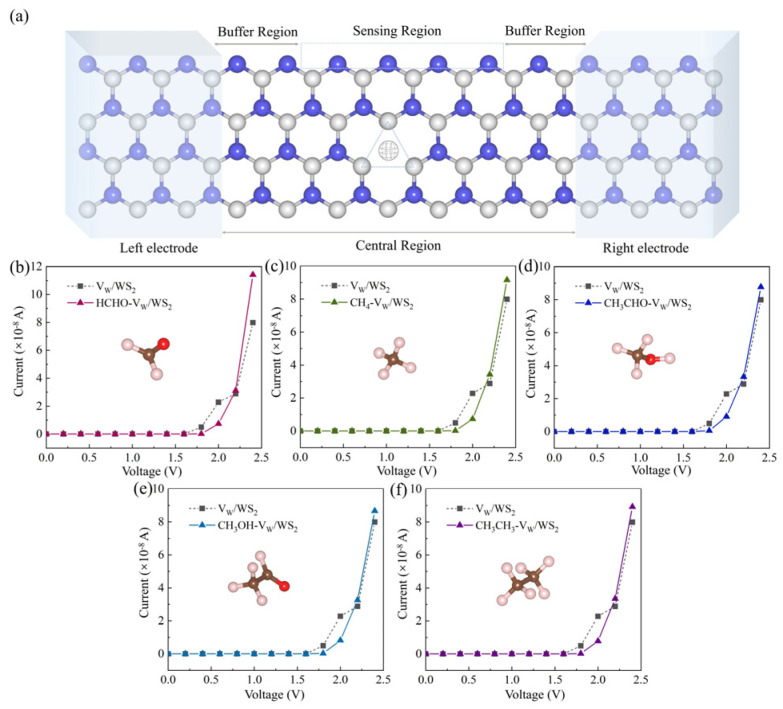
Device model for V_W_/WS_2_ adsorption systems of (**a**) and the I–V curve of (**b**) HCHO, (**c**) CH_4_, (**d**) CH_3_CHO, (**e**) CH_3_OH, and (**f**) CH_3_CH_3_ adsorbed on V_W_/WS_2_.

The relationship between *W* and the sensitivity of chemical sensors is mainly reflected in the change in electronic structure caused by the interaction between materials and target molecules. This relationship is very important for understanding the working mechanism of the sensor, optimizing the sensor’s performance, and designing a more selective and sensitive sensor. Additionally, we computed the *W* of five adsorption systems and the V_W_/WS_2_ [44]. Δ*W* represents the change in the *W* after adsorption, as seen in Table 2. The Δ*W* of the CH_4_-V_W_/WS_2_ (0.005 eV) and CH_3_CH_3_-V_W_/WS_2_ (0.008 eV) systems show a minor increase, and the CH_3_HO-V_W_/WS_2_ and CH_3_OH-V_W_ systems show a decrease [45,46]. The *W* clearly increases for the HCHO-V_W_/WS_2_ system, and the change value is 0.205 eV, which is noticeably larger than for other adsorption systems. This indicates that V_W_/WS_2_ has high sensitivity and selectivity for HCHO and that HCHO will interact with V_W_/WS_2_ more readily when it is in contact with experimental gas. Device models can be used to predict the performance of electronic devices, including I–V characteristic, power consumption, and speed [47]. We constructed device models, as Figure 11a illustrates. The gadget is separated into a center scattering zone and left and right electrodes. The buffer layer can adjust the charge distribution in the device and prevent excessive charge from accumulating in a certain area, thus maintaining the stability and reliability of the device. The buffer layer can also block the diffusion of external impurities, protect the internal purity of the device, and reduce the negative impact on the performance of the device, so, at the intersection of the electrodes and the central scattering region, we inserted three buffer layers to prevent any interference of the device center [48]. We utilized Materials studio software to compute the I–V characteristic of five adsorption systems and V_W_/WS_2_, which were represented by the I–V curve, so that we could clearly see the change in conductivity. The I–V curve is displayed in Figure 11b–f, where the gray lines in each figure correspond to the V_W_/WS_2_ I–V curve. We can observe that the current starts to increase when a bias voltage of 1.8 V is applied [49]. Compared with the I–V curve of BC_6_N adsorption systems [33], the slope change of the I–V curve after V_W_/WS_2_ adsorbed gas molecules is more obvious, and the conductivity change is more sensitive when detecting gas molecules. Figure 11b shows us that hardly any current passes through HCHO-V_W_/WS_2_ when the bias voltage is less than 1.8 V. The current increases dramatically when the applied bias voltage rises above the threshold value (1.8 V). The current of 1.14 × 10^−7^ A is reached at 2.4 V, which is much greater than that of V_W_/WS_2_ (7.99 × 10^−8^ A). Following adsorption, V_W_/WS_2_ is clearly sensitive to HCHO, as seen by the sharply altered I–V curve, higher slope (under the bias voltage of 1.8 V~2.4 V), and clearly increased conductivity. The I–V curves of the four types of adsorption systems in Figure 11c–f have altered somewhat when compared to the I–V curve of the V_W_/WS_2_. By examining the slope, we can also observe that V_W_/WS_2_ has the maximum sensitivity to HCHO, as seen by the I–V curves of the five adsorption systems (which is compatible with the *W* calculation results). It can also demonstrate the selectivity of the sensor, allowing us to differentiate HCHO from other gases through the observation of the conductivity shift. To validate the work function and I–V curve results, we computed V_W_/WS_2_ sensitivity at 1.8 V, 2.0 V, 2.2 V, and 2.4 V bias voltages [50,51]. Table 3 displays the results of the calculation [52]. The sensitivity of V_W_/WS_2_ to HCHO, CH_4_, CH_3_HO, CH_3_OH, and CH_3_CH_3_ is 98.1%, 96.7%, 88.9%, 94.9%, and 96.0%, respectively, when a bias voltage of 1.8 V is applied to the adsorption systems. This indicates that V_W_/WS_2_ has high sensitivity to these five gases at a voltage of 1.8 V. The sensitivity of Pt-NiS_2_ to HCHO is −99.2% [53], and the sensitivity of V_W_/WS_2_ to HCHO is −98.1% under 1.8 V, so it is necessary to study further. Significantly higher than that of other adsorbed gas molecules, the sensor’s sensitivity to HCHO is 108.0% and 42.9% at 2.2 V and 2.4 V, respectively [54,55,56]. This suggests that V_W_/WS_2_ can selectively detect HCHO, and the calculated results are in agreement with those of *W* and the I–V characteristic.

## 4. Conclusions

This research uses a series of simulations to study the gas sensitivity of V_W_/WS_2_ to HCHO, CH_4_, CH_3_HO, CH_3_OH, and CH_3_CH_3_. Initially, we used first-principles calculations to determine the system’s electronic properties. According to the DOS, HCHO contributes more than other gas molecules to an energy level close to the Fermi level. The HCHO-V_W_/WS_2_ *B* is the biggest (0.104 e). The system’s *W* was then computed, and the HCHO system’s *W* clearly rose. We also calculated the I–V characteristic. It was observed that in the case of adsorbed HCHO, the corresponding conductivity increased most dramatically and the slope increased most noticeably once the bias voltage rose beyond the threshold voltage. Therefore, V_W_/WS_2_ can solve the cross-sensitivity and other defects of general chemical sensors, and then develop into HCHO chemical sensors with high sensitivity and selectivity, which can be further integrated into flexible wearable devices to realize real-time monitoring of individual health and environmental factors.

## Figures and Tables

**Table 1:** The *E*_ad_, the *H*, band gap (*E*_g_), *B*, and *W* for WS_2_, and HCHO, CH_4_, CH_3_CHO, CH_3_OH, and CH_3_CH_3_ adsorbed on WS_2_.

Configuration	*E*_ad_ (meV)	*H* (Å)	*E*_g_ (eV)	*B* (e)	*W* (eV)	Δ*W* (eV)
WS_2_	-	-	1.775	-	5.39	
HCHO-WS_2_	196.72	2.94	1.772	0.107	5.72	0.337
CH_4_-WS_2_	149.55	2.80	1.772	0.092	5.53	0.140
CH_3_CHO-WS_2_	233.05	2.78	1.773	0.088	5.01	−0.374
CH_3_OH-WS_2_	206.27	2.49	1.773	0.091	5.15	−0.232
CH_3_CH_3_-WS_2_	246.02	2.52	1.773	0.089	5.53	0.146

**Table 2:** The *E*_ad_, *H*, *E*_g_, and *B* for HCHO, CH_4_, CH_3_CHO, CH_3_OH, and CH_3_CH_3_ adsorbed on V_W_/WS_2_.

Configuration	*E*_ad_ (meV)	*H* (Å)	*E*_g_ (eV)	*B* (e)	*W* (eV)	Δ*W* (eV)
V_W_/WS_2_	-	-	0.379	-	5.432	-
HCHO-V_W_/WS_2_	−207.8	2.92	0.340	0.104	5.637	0.205
CH_4_-V_W_/WS_2_	−144.5	2.79	0.341	−0.011	5.437	0.005
CH_3_CHO-V_W_/WS_2_	−188.2	2.65	0.373	0.090	4.993	−0.439
CH_3_OH-V_W_/WS_2_	−207.9	2.67	0.372	0.087	5.069	−0.363
CH_3_CH_3_-V_W_/WS_2_	−236.9	2.51	0.367	−0.016	5.440	0.008

**Table 3:** The sensitivity (%) of the sensor based on V_W_/WS_2_ with left and right electrodes toward HCHO, CH_4_, CH_3_CHO, CH_3_OH, and CH_3_CH_3_ at bias voltage of 1.8 V, 2.0 V, 2.2 V, and 2.4 V. Negative (positive) sensitivity means that the current of the sensor dropped (enhanced) after interaction with the adsorption.

Bias Voltage (V)	HCHO	CH_4_	CH_3_CHO	CH_3_OH	CH_3_CH_3_
1.8	−98.1	−96.7	−88.9	−94.9	−96.0
2.0	−67.7	−67.9	−60.0	−64.1	−65.8
2.2	+108.0	+18.9	+15.3	+13.2	+16.2
2.4	+42.9	+14.6	+9.75	+8.46	+11.7

## Data Availability

Data are contained within the article.
